# Robot-assisted gait training: more randomized controlled trials are needed! Or maybe not?

**DOI:** 10.1186/s12984-022-01037-z

**Published:** 2022-06-08

**Authors:** Rob Labruyère

**Affiliations:** 1 Swiss Children’s Rehab, University Children’s Hospital Zurich, Mühlebergstrasse 104, 8910 Affoltern am Albis, Switzerland; 2 Children’s Research Center, University Children’s Hospital Zurich, University of Zurich, Zurich, Switzerland

**Keywords:** Lokomat, Machine learning, Device settings

## Abstract

I was encouraged by the recent article by Kuo et al. entitled “Prediction of robotic neurorehabilitation functional ambulatory outcome in patients with neurological disorders” to write an opinion piece on the possible further development of stationary robot-assisted gait training research. Randomized clinical trials investigating stationary gait robots have not shown the superiority of these devices over comparable interventions regarding clinical effectiveness, and there are clinical practice guidelines that even recommend against their use. Nevertheless, these devices are still widely used, and our field needs to find ways to apply these devices more effectively. The authors of the article mentioned above feed different machine learning algorithms with patients’ data from the beginning of a robot-assisted gait training intervention using the robot Lokomat. The output of these algorithms allows predictions of the clinical outcome (i.e., functional ambulation categories) while the patients are still participating in the intervention. Such an analysis based on the collection of the device’s data could optimize the application of these devices. The article provides an example of how our field of research could make progress as we advance, and in this opinion piece, I would like to present my view on the prioritization of upcoming research on robot-assisted gait training. Furthermore, I briefly speculate on some drawbacks of randomized clinical trials in the field of robot-assisted gait training and how the quality and thus the effectiveness of robot-assisted gait training could potentially be improved based on the collection and analysis of clinical training data, a better patient selection and by giving greater weight to the motivational aspects for the participants.

More than 20 years ago, with the development of the gait trainers Lokomat (Hocoma AG, Volketswil, Switzerland) [1] and the Gaittrainer GT I (Reha-Stim, Berlin, Germany) [2] in the year 2000, the era of stationary robot-assisted gait training in neurorehabilitation began. These devices promised to bring revolutionary possibilities for patients and therapists. From a theoretical perspective, the potential to improve patient training was thought to be great because these devices operated according to the key principles of motor learning: many step repetitions, the possibility to make errors in a controlled fashion, and the visual feedback based on data collected by the device itself [3]. For therapists, the concept promised improvements in physical load compared to bodyweight-supported treadmill training and a high individualization to the patient due to the ability to adjust various biomechanical and training parameters. However, more than two decades of time and product development later, the many hopes may have been overstated. There have been many conflicting results regarding these devices’ effectiveness compared to non-robotic treadmill training, and already early on, large trials concluded that robot-assisted gait training is not superior to conventional physiotherapeutic interventions when it comes to reducing motor impairment [4, 5]. This did not significantly change moving forward, which has led to the publication of conflicting clinical practice guidelines for stroke, traumatic brain injury, and spinal cord injury that cover the recommendation spectrum from *do *to* don’t do* [6–12].

Nevertheless, other similar devices have come to market, and the robots mentioned above underwent further development based on the growing knowledge in the field of gait rehabilitation [13, 14]. From the author’s perspective, the publication of clinical practice guidelines recommending against the use of stationary robot-assisted gait trainers does not appear to have had a dramatic negative impact on the sales performance of the manufacturers of such devices. They focus on improving their devices regarding key elements of neural stimulation: Active patient participation through motivating virtual reality applications, meaningful real-time feedback from the device, and the incorporation of new controllers and hardware parts that increase degrees of freedom. So, these devices should be more effective compared to their predecessors; however, also current studies on robot-assisted gait training still report non-superior results on effectiveness compared to non-robotic gait training [9], and many of those studies end with a sentence similar to: “More large randomized controlled trials are needed”.

There are many possible reasons why robot-assisted gait training did not perform as hoped 20 years ago.

- Perhaps these devices are simply not yet at a good enough technological level to outperform conventional therapy, and another big leap in development is necessary to produce the according positive scientific results. This is reflected in the fact that current devices usually do not enable patients to reach a sufficient cardiovascular training intensity [15–17]. Training at high gait speeds is not possible with sufficient quality due to the weight and the associated inertia of these devices.
- Perhaps, the influence of neurorehabilitation on the recovery of the locomotor system after substantial damage is limited (*proportional recovery rule*, [18]), making it difficult for a “new” intervention to outperform conventional therapy without differentiating between “fitters” and “non-fitters”. Often, the aim of clinical trials is to show the superiority of robot-assisted gait training over conventional approaches [8], but would it not make sense to look at these devices as a tool to reach specific training goals instead of a jack of all trades-device that outperforms alternative approaches?
- Perhaps, the actual clinical implementation is as big a problem as the technology itself. If these devices are merely tools, it is essential how therapists use them and that these therapists have an appropriate background. If one eats soup with a fork, it is the failure of the user or his/her education, not of the tool itself. Indeed, there are indications that these devices are sometimes actually used almost like “soup forks” in everyday clinical life. I have encountered therapy sessions in different facilities where these devices were misused by simply letting patients be passively walked without active guidance from trained therapists, by applying the same settings to all patients, by applying these devices to patients who can walk without walking aids, by using the device without proper biomechanical alignment to the individual patient, or by applying the device in the absence of a specific training goal. Such use of these tools certainly does not lead to significant systematic training progress and may even be detrimental.
- Perhaps, the increasing number of different settings these devices offer, without clear guidelines on how to use them, also contribute to a suboptimal application. As an example, the gait robot Lokomat currently theoretically offers 119′070 possible setting combinations if we only look at the primary device parameters (gait speed: 27 levels (0.5–3.2 km/h in steps of 0.1 km/h), bodyweight support: 21 levels (100–0% in steps of 5%), robotic support: 210 levels (guidance force and path control combined). Even if many of those combinations clinically do not make sense, it is still a vast search space. Accordingly, a striking common feature of robot-assisted gait training studies is the lack of adequate reporting of the selected settings of the device parameters (like bodyweight support, robotic support, treadmill/walking speed, etc.) and strategies in choosing those settings [19]. This lack prevents readers/therapists from transferring knowledge from such studies to their own therapy.

These could all be possible reasons for the lack of superiority of robot-assisted gait trainers over conventional therapies. One might therefore ask the question if we expect that “further large definitive pragmatic phase 3 trials” that “address specific questions about the most effective frequency and duration of electromechanical‐assisted gait training”, as proposed in the conclusion of the update of the Cochrane review authored by Mehrholz et al. [9], really help us to advance the field. Could it even be that the way researchers study these devices in randomized controlled trials contributes to the situation we find ourselves in? A randomized controlled trial is a prospective, comparative, quantitative study performed under controlled conditions with random allocation of interventions to comparison groups. Evidence stemming from RCTs is considered to be of the highest quality because an RCT aims to reduce bias systematically [20]. However, such a study will only deliver meaningful results if the investigated device is optimally applied. Otherwise, we are not investigating the device’s effectiveness but rather that of the paradigm used by the therapist applying the device. And there are many RCTs where robot-assisted gait training did not appear to be optimally applied. In our recent review on the reporting quality of Lokomat studies [19], we found several RCTs that did not change one or even several of the Lokomat settings over the whole course of the study [21–26], which might be an indication that the customization options of the device have not been fully exploited. So, as long as RCTs do not explicitly address the influence of different device settings on short-term improvements in patients, RCTs should not be the tool of choice to investigate the device’s effectiveness.

Our field is currently not yet in the position where we fully understand how robot-assisted gait trainers work and how we can optimally apply them. Therefore, our research should focus more on better understanding and helping therapists apply these devices and less on comparing the effects of suboptimal use of these devices with those of alternative therapies. As mentioned above, therapists can choose from many different settings, like bodyweight support, robotic support, or treadmill/walking speed, and the effects of these settings interact with each other [27, 28]. Over the last years, studies have started looking into this subject, and there are clear indications that the choice of the parameters might influence the device’s effectiveness. To name a few examples, van Kammen et al. found higher EMG amplitudes with decreased guidance force and increased walking speed [28]. Similarly, Aurich et al. were able to show that muscle activation patterns during robot-assisted gait training normalize with lower robotic support [13]. A beneficial effect of gait speed on the patients’ activity has also been found by Krewer et al. [29]. There are several reasons why parameter settings might be important: (1) The key success factors of motor learning mentioned above can be directly influenced by the parameters of robot-assisted gait trainers. Therefore, the choice of parameter settings directly influences the quality and intensity of the training. (2) Each clinic or even each therapist has its own preferences for setting up the device, and it is rarely done in a systematic, evidence-based way. This is expressed in, but also due to, the variety of parameter settings reported in the literature [19]. As such, heterogeneity in choosing these parameter settings might lead to heterogeneity in the outcome. (3) On top, the parameter settings during RCTs usually do not reflect everyday clinical life as they tend to be much more strictly controlled. Therefore, these RCTs might provide an inadequate estimate of the actual effectiveness of the investigated device. (4) Device parameters are not independent; cross-sectional studies could show their interplay [27, 28]. Therefore, it is essential to bring these parameters into the focus of therapists and researchers. There needs to be consistent reporting of the parameter settings in scientific literature, and we need to learn much more about the influence of the parameters on each other and on the effectiveness of the training. Furthermore, the search for appropriate short-term markers of training effectiveness also plays a significant role in advancing our field [30–32].

Given that the Lokomat produces extensive data outputs from each training session, and based on the developments in data analysis, it seems a logical next step to look for patterns in the data already available from the robotic gait trainers. That is precisely what Kuo et al. did in their paper, recently published in the Journal of NeuroEngineering and Rehabilitation [33]. The authors used the Lokomat settings’ data (bodyweight support, gait speed, and robotic assistance) from a cohort of 91 patients from the beginning to predict the functional ambulation categories at the end of the Lokomat intervention. They tried several machine learning algorithms to extrapolate the data to investigate factors associated with patient recovery. Their results show that machine learning models fed with early-stage Lokomat data can predict the clinical outcome of these patients at a later stage. Even though the study has clear limitations (e.g., small cohort, collection time points of Lokomat data, relatively few data from each participant, methodological duplication of datasets, patients selection), this study represents a proof-of-concept for the application of machine learning to clinical data from Lokomat training sessions and demonstrates the potential of this approach. It might well be that the course of the chosen parameter settings during a robot-assisted gait training intervention combined with a quantification of the therapy goals might contain much more relevant information than two clinical assessments at single time points before and after the intervention. It could also provide a deeper insight into the ongoing discussion on responders vs. non-responders [34–37].

Therefore, it might make sense to focus our research on obtaining more information on how good therapists choose device parameter settings and how these settings interact with each other and with the patients’ clinical phenotype rather than on continuing randomized clinical trials from which we cannot draw meaningful conclusions. Such information could eventually be used to support the therapists in adjusting the device parameters during the intervention with the help of algorithms. Such an “algorithmization” of the parameter selection would allow rigorous scientific testing of robot-assisted gait training while ensuring patient-optimal customization of the intervention. Ultimately, that would most likely also have implications for the much-debated effectiveness of this therapy form.

The key prerequisites to the meaningful clinical use of stationary robotic gait trainers lie in the optimal patient selection [6], the optimal adaptation of the device and the settings to the individual situation and the training goal of the patient [13, 38], and the active contribution of the patient [3, 28, 39] that must be guided by a well-trained therapist [13, 40, 41]. Under these circumstances, these devices can be a valuable component of the rehabilitation process for patients [36]. However, as scientists, clinicians and manufacturers, we bear the responsibility to ensure that the prerequisites mentioned above are met; otherwise, we are robbing patients of valuable time. Therefore, as long as these devices are in our hospitals and rehabilitation centers, let us ensure they are put to optimal use.

## Data Availability

Not applicable.
